# Functional materials design: octahedral tilts in hybrid *n* = 1 Ruddlesden–Popper phases

**DOI:** 10.1107/S2052252523005559

**Published:** 2023-06-28

**Authors:** Emma E. McCabe

**Affiliations:** a Durham University, Lower Mountjoy, South Road, Durham, DH1 3LE, United Kingdom

**Keywords:** perovskites, symmetry mode analysis, organic–inorganic hybrid materials, Ruddlesden–Popper structure

## Abstract

The exciting properties offered by hybrid perovskite-related materials have motivated Liu *et al.* [(2023). *IUCrJ*, **10**, 385–396] to explore the crystallography of hybrid *n* = 1 Ruddlesden–Popper phases. Their investigation explores the structures (and symmetries) expected to result from typical distortions and gives design strategies to target specific symmetries.

The importance of hybrid perovskites and more recently their layered analogues [*e.g.* the high efficiency and stable Ruddlesden–Popper (RP) phases (Stoumpos *et al.*, 2016 ▸; Tsai *et al.*, 2016 ▸)] is undisputed with significant research resources dedicated to these functional materials. Recently several excellent studies of the structural chemistry of hybrid layered perovskite-related materials have been published (Saparov & Mitzi, 2016 ▸; Mao *et al.*, 2019 ▸) including an in-depth exploration by McNulty and Lightfoot of several families of layered hybrid perovskite-related materials (McNulty & Lightfoot, 2021 ▸). Understanding the precise structure (and symmetry) of these systems is essential for designing and optimizing functional materials, but until recently, there have been few studies on specific families: Li *et al.* provide an interesting comparison of hybrid Dion–Jacobson materials (Li *et al.*, 2019 ▸) with their all-inorganic analogues (Aleksandrov & Bartolomé, 2001 ▸), but the *n* = 1 RP family has not received a systematic crystallographic investigation until now. In this issue of **IUCrJ** , Liu *et al.* take on this challenge with gusto, mapping out the structures expected for the hybrid *n* = 1 RP materials (Liu *et al.*, 2023 ▸). This builds on the excellent work of McNulty & Lightfoot (2021 ▸), but by focusing on only a single family, Liu *et al.* can identify specific drivers for tilts and structure types, and even go some way to providing a blueprint for designing hybrid *n* = 1 RP phases with targeted symmetries.

In order to fully appreciate the authors’ work, it is useful to briefly consider the wider field of structural chemistry of perovskites (and related phases). The mineral perovskite, CaTiO_3_, named after Count Perovski, gives its name to this structure type and well known family of materials (Attfield *et al.*, 2015 ▸). Perovskites have long been an important research focus, from the all-inorganic phases [*e.g.* ferroelectric BaTiO_3_ (Megaw, 1952 ▸)] to the recent ‘hybrid’ perovskites [*e.g.* CH_3_NH_3_PbI_3_ (Kojima *et al.*, 2009 ▸)] known for their impressive photovoltaic properties (NREL, 2023 ▸). With the general formula *ABX*_3_ (*A*, *B* are cations; *X* are anions), the typical perovskite structure can be described in terms of cubic-close-packed *AX*_3_ layers with smaller *B* cations in between (West, 2014 ▸) to give the familiar three-dimensional network of corner-linked *BX*_6_ octahedra [Fig. 1 ▸(*a*)]. What makes the perovskite family of materials so important is their compositional flexibility: this allows the design of functional perovskite materials with a huge range of properties.

The relatively simple topology of corner-linked *BX*_6_ shown in Fig. 1 ▸(*a*) belies the rich structural complexity of this family: the arisotype structure is of 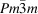 symmetry, but few materials adopt this ideal structure and several types of distortion (driven by geometric or electronic factors) are possible (Salje *et al.*, 1989 ▸). An important type of distortion involves rotation of essentially rigid *BX*_6_ octahedra (Glazer, 1972 ▸; Howard & Stokes, 1998 ▸; Woodward, 1997 ▸) to optimize bonding for the various ions. The resulting change in structure (and symmetry) has an enormous impact on properties and so tuning these rotations has been a powerful method of optimizing properties.

Alongside the perovskite with its three-dimensional connectivity of *BX*_6_ octahedra, several families of perovskite-related materials with layered structures are known, including the RP (Ruddlesden & Popper, 1957 ▸, 1958 ▸) and Aurivillius families (Aurivillius, 1950*a* ▸,*b* ▸, 1952 ▸) [Figs. 1 ▸(*b*), 1(*c*)]. The connectivity of *BX*_6_ octahedra is maintained in two dimensions, but perovskite blocks are separated along the third direction by either *AX* rocksalt layers (for the RP phases) or by fluorite-like [Bi_2_O_2_]^2+^ layers for the Aurivillius phases. As for the *ABX*_3_ perovskites, these phases readily undergo distortions from their aristotype structures (of *I*4/*mmm* symmetry) involving rotations of the *BX*_6_ octahedra. These have been systematically explored in the context of all-inorganic materials (Aleksandrov & Bartolome, 1994 ▸; Aleksandrov & Bartolomé, 2001 ▸; Hatch & Stokes, 1987 ▸; Hatch *et al.*, 1989 ▸) and this understanding has been key in designing complex functional materials (Pitcher *et al.*, 2015 ▸; de Araujo *et al.*, 1995 ▸; Oh *et al.*, 2015 ▸).

The hybrid analogues adopt structures with the same topology, comprising networks of corner-linked *BX*_6_ octahedra (such as the lead halide systems), but with molecular cations on the *A* site, such as ammonium and methyl­ammonium. The hybrid systems have similar structural degrees of freedom to the all-inorganic materials but with added complexity due to the shape and different bonding characteristics of the molecular *A* cations (Saparov & Mitzi, 2016 ▸; Mao *et al.*, 2019 ▸; McNulty & Lightfoot, 2021 ▸). Understanding the structures and symmetries of these hybrid layered perovskite-related systems, with significant dispersion forces and hydrogen bonding distinguishing them from their all-inorganic counterparts, will be key for designing optimized functional materials.

Liu *et al.* focus on the *n* = 1 RP [Fig. 1 ▸(*b*)] and their work (Liu *et al.*, 2023 ▸) will be valuable to those studying both on hybrid and all-inorganic materials. Their approach is to use the extremely powerful web-based *ISODISTORT* software (Stokes *et al.*, 2006 ▸; Stokes *et al.*, 2022 ▸) to identify the symmetry-adapted distortion modes to describe possible rotations of *BX*_6_ octahedra about both in-plane and out-of-plane axes. The authors then determine the resulting space groups and basis vectors that allow these degrees of freedom (individual tilts and combinations of tilts). Crucially this work builds on earlier studies by including in-phase tilts about an in-plane axis [ 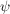 tilts, in Aleksandrov notation (Aleksandrov *et al.*, 1987 ▸)]. These tabulated results alone will be an important resource for those working on the structural characterization of *n* = 1 RP (and Aurivillius) phases.

Surveying structural databases (the Cambridge Structural Database and the Inorganic Crystal Structure Database) allowed the authors to identify common structures and tilt patterns in hybrid *n* = 1 RPs and to make comparisons with the all-inorganic analogues. Their findings highlight the importance of hydrogen bonding and dispersion forces – both in explaining the relative energy scales (and therefore temperatures) of the distortions, and in understanding the different tilt patterns observed.

A key highlight for me are the findings regarding loss of inversion symmetry in these *n* = 1 materials. Both out-of-phase tilts about an in-plane axis ( 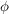 tilts) and tilts about an out-of-plane axis ( 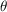 tilts) do not break inversion symmetry – either alone, or in combination [as emphasized by McNulty & Lightfoot (2021 ▸)] giving centrosymmetric structures (see Table 1 in Liu *et al.*). The *n* = 1 Aurivillius phases provide all-inorganic examples of this, with the common tilt pattern 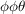 / 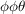 giving centrosymmetric structures of *Pbca* symmetry:

(*a*) with only tilts [*e.g.* Bi_2_NbO_5_F (McCabe *et al.*, 2007 ▸)], the structure is of *Pbca* symmetry;

(*b*) additional polar displacements are needed to break inversion symmetry [*e.g.* to *Pca*2_1_ symmetry with in-plane polarization as for Bi_2_WO_6_ (Knight, 1992 ▸, 1993 ▸) and Bi_2_MoO_6_ (Teller *et al.*, 1984 ▸); see also the clear discussion by McNulty & Lightfoot (2021 ▸), and the symmetry map for Bi_2_TiO_4_F_2 _(Giddings *et al.*, 2021 ▸)].

This contrasts with the *n* = 2 RP and Aurivillius phases for which equivalent tilt patterns may break inversion symmetry (Aleksandrov & Bartolome, 1994 ▸) [*e.g. A*_2_LaTaTiO_7_ (Mallick *et al.*, 2021 ▸), Sr_3_Zr_2_O_7_ (Yoshida *et al.*, 2018 ▸)].

On the other hand, if in-phase ( 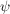 ) tilts about an in-plane axis are included, inversion symmetry is often broken (see Table 2 in Liu *et al.*). There are few examples of systems including 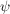 tilts. The authors explain this in terms of the different cation environments that result, and suggest cation-ordering strategies to design such non-centrosymmetric systems among the hybrid *n* = 1 RP phases.

This systematic crystallographic study of the possible tilt combinations and structures for *n* = 1 RP phases will be a key reference for those working on both hybrid and on all-inorganic systems. It will be particularly exciting to see researchers apply this understanding of the molecular *A* cation bonding to realize specific tilt combinations (and symmetries) as they seek to prepare new functional materials with optimized properties.

## Figures and Tables

**Figure 1:**
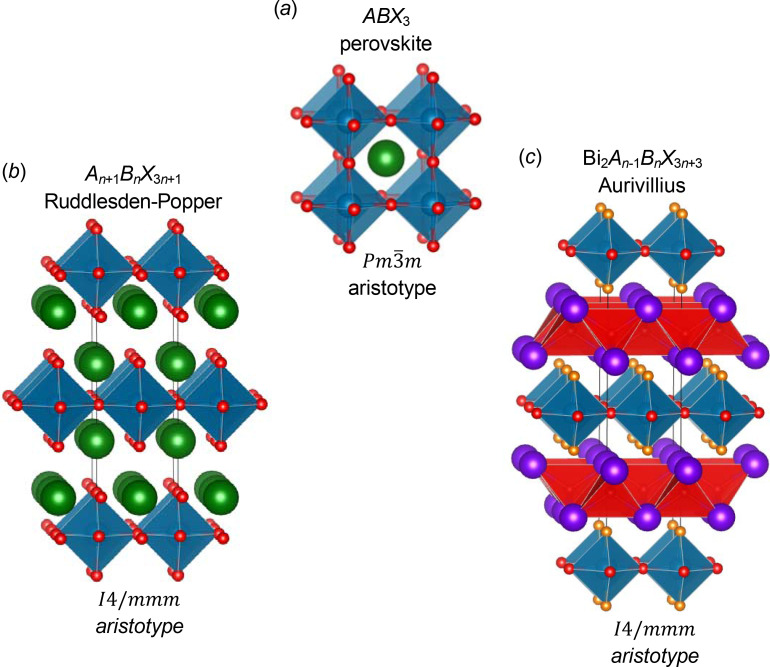
In this figure (*a*) shows the ideal aristotype structure of the cubic perovskite with general formula *ABX*_3_; (*b*) shows the ideal structure of an *n* = 1 RP phase (*e.g.* Sr_2_TiO_4_) and (*c*) shows the aristotype structure of an *n* = 1 Aurivillius phase [*e.g.* Bi_2_TiO_4_F_2_ (Needs *et al.*, 2005 ▸)]. *A*, *B* and *X* ions are shown in green, blue and red, respectively with *BX*_6_ octahedra shown in blue and the [Bi_2_O_2_]^2+^ fluorite-like layers in the Aurivillius structure shown in red.
